# Numerical Simulation of Fluid Shear Stress Distribution in Microcracks of Trabecular Bone

**DOI:** 10.1155/abb/5634808

**Published:** 2025-01-15

**Authors:** Yan Gao, Sen Zhao, Ailing Yang

**Affiliations:** ^1^Capital University of Physical Education and Sports, Institute of Artificial Intelligence in Sports, Beijing 100191, China; ^2^Beijing Institute of Technology, School of Aerospace Engineering, Beijing 100081, China

**Keywords:** distribution, fluid shear stress, microcrack, numerical simulation, trabecular bone

## Abstract

Bone is one of the hardest tissues in the human body, but it can undergo microcracks under long-term and periodic mechanical loads. The Newton iterative method was used to calculate the steady state, and the effects of different inlet and outlet pressures, trabecular gap width and height, and microcrack's depth and width on the fluid shear stress (FSS) were studied, and the gradient of FSS inside the microcrack was analyzed. The results show that the pressure difference and trabecular gap heigh are positively correlated with the FSS (the linear correlation coefficients *R*^2^ were 0.9768 and 0.96542, respectively). When the trabecular gap width was 100 μm, the peak of FSS decreased by 28.57% compared with 800 and 400 μm, and the gradient of FSS inside the microcrack was 0.1–0.4 Pa/mm. This study can help people more intuitively understand the internal fluid distribution of trabecular bone and provide a reliable theoretical basis for the subsequent construction of gradient FSS devices in vitro.

## 1. Introduction

**Figure 1:**
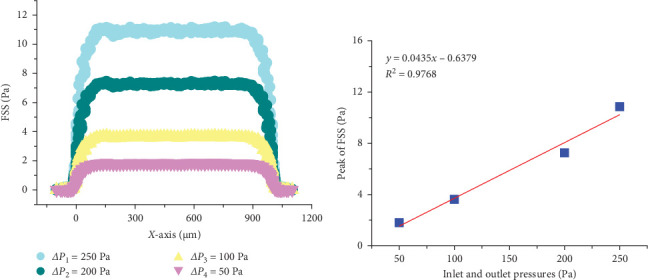
The effect of inlet and outlet pressure differences on fluid shear stress in the bone trabecular interstices. (A) Fluid shear stress along the *X*-axis under different inlet and outlet pressures. (B) The impact of varying inlet and outlet pressure differences on peak fluid shear stress in the bone trabecular interstices.

As a special biomechanical material, bone tissue possesses functional adaptability, able to change with variations in the magnitude or direction of external forces. The orientation of bone trabeculae aligns with the principal stress trajectories, and the patterns of skeletal structural evolution resulting from these mechanical factors are also known as “Wolff's law” [1]. In a physiological state, bone formation led by osteoblasts and bone resorption led by osteoclasts are in dynamic equilibrium. This adaptive structural adjustment process of bone is termed bone remodeling. When various factors disrupt the balance of bone remodeling, it can lead to various orthopedic diseases. For instance, compromised microstructure of bone tissue and decreased bone density are primary characteristics of osteoporosis (OP) [2]. This condition can lead to increased bone fragility and an elevated risk of fractures [3]. Researchers investigated how the material properties and geometric shapes of bone tissue can undergo functional adaptive changes in response to mechanical loads through the process of bone remodeling, using biomechanical simulations [4–6]. The previous research by the research group utilized the actual structure of the distal femoral trabecular bone in mice to establish a three-dimensional (3D) finite element model (Figure 1). The results of fluid–solid coupling numerical simulations indicated that the stiffness along the direction of physiological load was significantly higher than the stiffness perpendicular to the direction of physiological load [7]. “In the past 30 years, most research on predicting the evolution of bone structure based on the principles of cell mechanics has tended to focus on the bone formation processes dominated by mesenchymal stem cells, osteoblasts, and osteocytes.” Bentolila et al. [8] and others have long discovered that there is a colocalization relationship between bone remodeling areas and areas of microdamage within the bone. Specifically, bone resorption usually occurs near large-scale microdamage rather than near small-scale, diffusely distributed microdamage [9, 10]. “When the human body moves, the contraction forces from muscles along with gravity cause the bones to deform. The deformation of the bone matrix directly induces the attached cells to produce corresponding strain” [11, 12].

Before the 1970s, there was more focus on the impact of bone matrix deformation on the biological response of bone tissue cells. At that time, it was not believed that a hard tissue like bone would have fluid flow nor was it considered that bone cells could be affected by fluid shear stress (FSS). In 1977, Piekarski and Munro [13] took into account the presence of fluid in the porous structure of bone and proposed that mechanical loads could cause fluid flow within the bone. Subsequent research has shown that compared to matrix deformation [14], bone tissue cells are more responsive to fluid stimulation [15]. For example, osteoclast precursor cells can sense the stimulation of FSS and are more inclined to migrate in the direction of the fluid under the stimulation of 10 dyne/cm^2^ FSS [16]. This directional migration is regulated by the calcium ion signaling pathway [17, 18]. Fluid stimulation of osteoblasts at different stages of differentiation (murine osteocyte-like cell line, murine embryo osteoblast precursor cell line, and murine preosteocyte like cell line) has been found to increase the frequency of calcium oscillations in later stages of bone formation [19, 20]. Additionally, matrix deformation can also cause changes in the volume of the pores within the bone, which in turn generates fluid pressure gradients at different locations within the pores, leading to fluid flow. Previous research by our group has shown that, in a gradient fluid environment, osteoclast precursor cells do not migrate in the direction of fluid flow but instead migrate directionally toward areas of lower stress [21, 22]. Therefore, it is crucial to focus on the distribution pattern of FSS in the microstructural regions of bone trabeculae, particularly the regulatory effects of local FSS on cellular biological responses, which have previously received little attention.

When the human body moves, the contraction forces from the muscles and gravity cause the bones to deform. The deformation of the bone matrix directly induces a corresponding strain in the cells attached to it. Additionally, the deformation of the matrix alters the volume of the cavities within the bone, which in turn creates a fluid pressure gradient at different locations within these cavities, causing fluid flow and ultimately generating FSS on the surface of the cells [23, 24]. It should be noted that as early as the 1990s, experiments in cellular biomechanics in vitro had demonstrated that compared to deformations of the extracellular matrix, bone tissue cells are more responsive to fluid stimulation [14, 15, 25]. Previous research has found that there is a colocalization relationship between bone reconstruction areas and bone microdamage zones; particularly, bone resorption typically occurs near large-scale microdamage rather than near small-scale microdamage that is dispersed [8–10]. Therefore, it is reasonable to speculate that osteoclast precursor cells sense the FSS gradient near the microinjury and migrate to the low FSS area inside the microinjury, gradually differentiate into mature osteoclasts, and finally initiate bone resorption.

Microcracks in bone are minute fractures within the bone matrix that occur as a result of mechanical stresses. These tiny fractures can be critical to bone health and remodeling. According to the mechanostat theory, bone adapts its strength in response to mechanical stress; microcracks may serve as signals for bone remodeling processes. When microcracks accumulate, osteocytes (bone cells) can detect these changes and initiate repair by signaling for osteoclastic resorption and osteoblastic bone formation [26]. This process is crucial for maintaining bone integrity and preventing larger fractures. However, excessive accumulation of microcracks can be detrimental. If the rate of microcrack formation exceeds the repair capacity of the bone, it can lead to bone fragility and increase the risk of fractures [27]. Advances in imaging techniques have significantly enhanced our ability to detect and quantify microdamage. Techniques such as microcomputed tomography (micro-CT) and scanning electron microscopy (SEM) are commonly used to observe and measure these microscopic fractures. These techniques provide insights into the pattern, density, and distribution of microcracks, which are crucial for understanding the mechanical properties of bone [28, 29].

Bone microfractures are critical to understanding the progression of various orthopedic conditions and the efficacy of treatments. Finite element analysis (FEA) has been instrumental in simulating these microfractures, providing insights that are difficult to obtain through experimental methods alone. Recent advancements in FEA techniques have significantly improved the resolution and accuracy of bone microfracture simulations. Research has shown that by incorporating realistic bone material properties and geometries, simulations can predict the location and progression of microfractures under different loading conditions. For instance, studies have utilized patient-specific models derived from high-resolution imaging techniques such as MRI and CT scans, which enhance the predictive capabilities of the simulations regarding fracture risk and bone strength deterioration [4, 6, 30]. Recent studies have utilized finite element modeling (FEM) to simulate fluid flow and shear stress in bone under various physiological conditions. These simulations help in understanding how alterations in bone architecture associated with diseases like OP can affect fluid flow and thus cellular activity. Zhao et al. [31] utilized a coupled method of fluid dynamics and solid mechanics to simulate the mechanical behavior and stress distribution of the caudal vertebrae under dynamic loading. By constructing a detailed 3D model of the caudal vertebrae and applying appropriate physical and material parameters, the research was able to thoroughly analyze the biomechanical responses of the caudal vertebrae under various dynamic loading conditions.

Recent studies have utilized FEM to simulate fluid flow and shear stress in bone under various physiological conditions [31–33]. However, the distribution of FSS near microdamage is difficult to be measured directly by experiment. In this paper, the microdamage model of bone trabeculae was established by finite element numerical simulation. The effects of different inlet and outlet pressures, gap width, gap height, microcrack depth, and microcrack height on the distribution of FSS were studied, and the FSS gradient at the microdamage location was analyzed. FSS in bones arises primarily due to physical activity, which induces pressure gradients leading to fluid movement through the bone's lacunocanalicular network. Unfortunately, what is the value of the pressure inside the trabecular bone, because this is difficult to measure directly by experiment.

However, there are currently no reports on whether the mechanical microenvironment within bone trabeculae affects the dynamic reconstruction process of bone tissue. Therefore, this study hypothesizes that this may be due to changes in the distribution of FSS between trabeculae following microdamage, leading to directed cell migration. Due to the lack of in vivo measurement techniques, it is difficult to understand the fluid distribution inside the trabeculae. This paper will use numerical simulation methods to construct a model of trabeculae with microdamage; to study the effects of different inlet and outlet pressures, trabecular gap width, gap height, microcrack depth, and microcrack width on the distribution pattern of FSS in the model; and to conduct an in-depth study of the FSS gradient distribution inside the cracks.

## 2. Materials and Methods

### 2.1. Establishment of a Simplified Numerical Model for the Microstructure of Trabecular Bone

**Figure 2:**
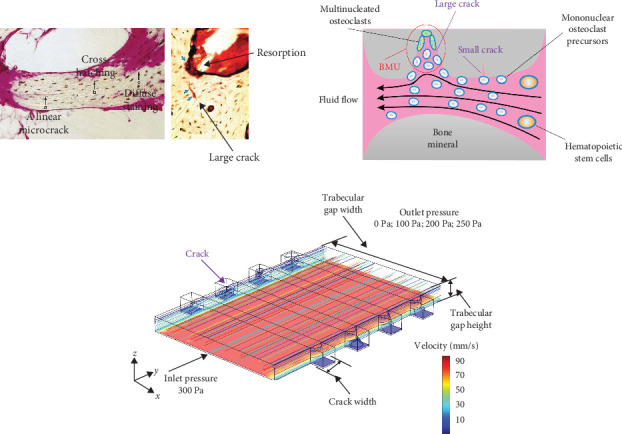
Microcracks in bone trabeculae. (A) Actual image of microcracks and bone resorption in bone trabeculae [34]. (B) Schematic diagram of the microcrack area in bone trabeculae. (C) Finite element model of microcracks in bone trabeculae.

There is evidence suggesting that bone resorption areas and microdamage within the bone are colocalized, indicating that bone resorption is not randomly distributed. These areas typically occur near larger microcracks rather than around diffuse microcracks [1] (Figure 2A,B). Due to the limitations of in vivo measurement techniques, directly obtaining the distribution of FSS within trabecular bone through experiments is challenging. Numerical simulations have emerged as an effective method to study microdamage in bone, offering a more intuitive understanding of FSS distribution within trabeculae. As shown in Figure 2C, a simplified numerical model of the trabecular bone microstructure has been established. The model parameters are detailed in Table 1. Indeed, the environment between trabeculae is characterized by complex and tortuous geometry. This paper uses the simplified model as the foundation for advancing this important area of research.

### 2.2. Computational Fluid Dynamic Simulation

The boundary conditions of the numerical model for the trabecular microstructure region, as developed in this study, are based on prior literature.

The internal environment of real trabecular bone is complex and dynamic, and the inlet and outlet conditions are referenced from previous studies. Physiologically, FSS in osteoblasts and other cells is reported to range from 0.8 to 3 Pa [38]. In earlier research, the FSS gradient in the flow cavity of a gradient parallel plate was calculated using pressure differences of 300, 200, and 100 Pa [21]. These pressure differences are comparable to those found in the lacunar–canalicular system within the bone, which ranges from 0.8 to 3 Pa [24]. In this study, the effects of different pressure differences at both ends of the trabecular bone model (250, 200, 100, and 50 Pa) on the FSS distribution in the microdamage region within the bone are evaluated through numerical simulations.

It is well-established that the viscosity coefficient of bone interstitial fluid is not constant but decreases progressively with increasing strain rate. Anderson et al. [39] assumed that the fluid characteristics within the bone lacunae and canaliculi are similar to saline, with a density of 997 kg/m^3^ and a dynamic viscosity of 0.855 mPa·s. Accordingly, in this study, the fluid density is set to 1000 kg/m^3^, and the dynamic viscosity is taken as the value for water at room temperature, 1 × 10^−3^ Pa·s. The trabeculae diameter is ~130 μm, with an average spacing of around 1000 μm [35]. The linear and cross-type microcracks in the trabecular bone are relatively large, ranging from 10 to 100 μm, which is comparable to the size of osteoclasts. Therefore, the depths and widths of the microcracks modeled in this study are set at 100, 50, 20, and 10 μm [36, 37]. The model used in this study employs the meshing function within the commercial software COMSOL to generate a 3D tetrahedral mesh. The fluid dynamics module is selected for steady-state analysis, assuming laminar flow, with calculations performed using the Newton–Raphson iterative method.

Finally, the model was meshed in COMSOL (Stockholm, Sweden), resulting in ~200,000 mesh elements for the finite element model of the microcracks. A convergence analysis was conducted to ensure mesh convergence.

## 3. Results

### 3.1. The Pressure Difference at the Entrance and Exit Is Positively Correlated With the Peak FSS in the Trabecular Bone Gap

This article calculates the effects of different pressure differences at both ends of the trabecular bone model (250, 200, 100, and 50 Pa) on the FSS distribution in the area of microdamage within the bone through numerical simulation. A pressure of 300 Pa was set at the inlet of the model with a trabecular gap of 100 μm. The results indicate that as the pressure difference between the entrance and exit increases, the distribution of FSS within the trabecular gap also increases. As can be seen from Figure 1A, the FSS near microdamage is lower than that in the trabecular gap under different entrance and exit pressure differences, and there is a positive correlation between the entrance and exit pressure difference and the peak FSS in the trabecular gap (Figure 1B). Therefore, this paper hypothesizes that during physical activity, changes in the frequency of movement cause variations in the flow rate of the interstitial fluid within the bone, yet the FSS inside the microcracks remains lower than in the gaps of the trabecular bone.

### 3.2. Reduced Width of the Bone Trabecular Interstices Results in Decreased Peak FSS

**Figure 3:**
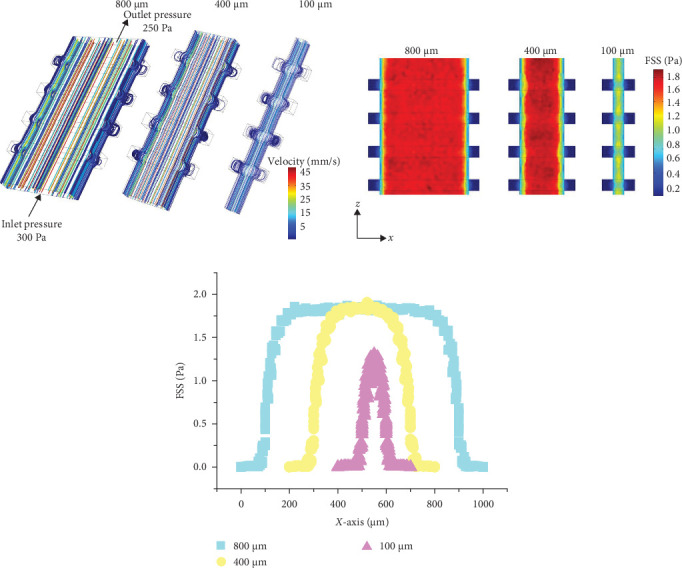
The impact of the gap width between trabecular bones on the distribution of fluid shear stress. (A) Finite element numerical models with different trabecular bone gap widths. (B) Top view of the fluid shear stress distribution in the numerical model of the trabecular bone microstructure. (C) Distribution of fluid shear stress along the *X*-axis at different trabecular bone gap widths.

This text establishes six different trabecular bone gap widths (1000, 800, 600, 400, 200, and 100 μm), as shown in Figure 3A. As the trabecular gap width decreases, the fluid velocity decreases, and the FSS along the *X*-axis exhibits a distribution pattern of initially increasing, followed by a stable period, and then decreasing, forming an inverted “U” shape. Additionally, as seen from Figure 3B, the FSS amplitude near microcracks is significantly lower than that within the trabecular gaps. Moreover, as the trabecular gap width decreases, the peak FSS within the gaps also decreases. When the gap width is reduced to 100 μm, the peak FSS within the trabecular gaps is about 28.57% lower than that at 800 μm, as shown in Figure 3C.

### 3.3. The Height of the Trabecular Bone Space Is Positively Correlated With FSS Within the Space, While the Depth and Width of the Cracks Do Not Affect the Shear Stress of the Fluid Within the Space

**Figure 4:**
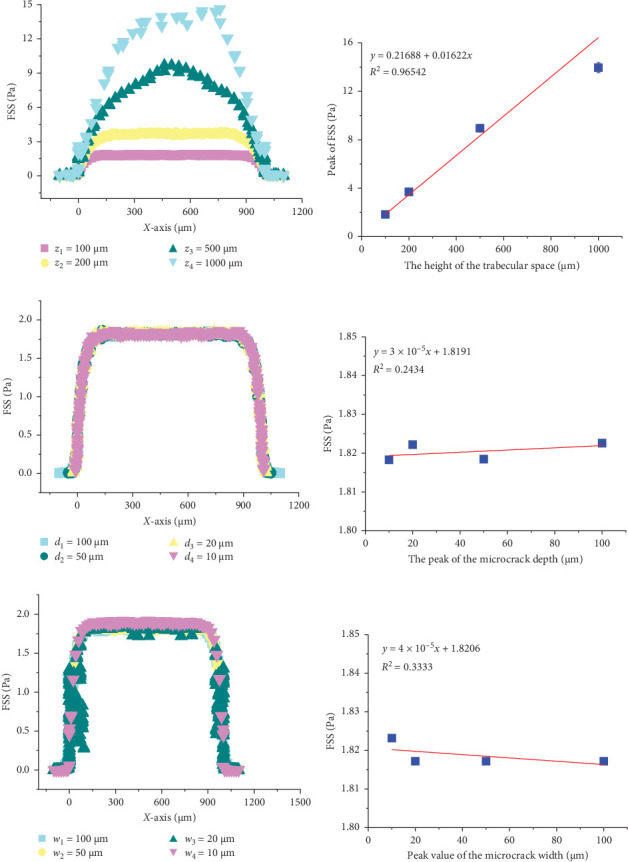
Analysis of the effects of trabecular bone gap height, microcrack depth, and width on fluid shear stress within the gap. (A) Simulation of fluid shear stress along the *X*-axis direction in trabecular bone gaps at different gap heights. (B) Peak fluid shear stress at different gap heights. (C) Simulation of fluid shear stress along the *X*-axis direction in trabecular bone gaps at different microcrack depths. (D) Peak fluid shear stress at different microcrack depths. (E) Simulation of fluid shear stress along the *X*-axis direction in trabecular bone gaps at different microcrack widths. (F) Peak fluid shear stress at different microcrack widths.

In addition to the relationship between trabecular bone space width and FSS demonstrated in the previous research results, this paper also discusses the impact of trabecular bone space height and microcrack depth and width on the FSS within the space. Initially, four trabecular bone space heights were set at 100, 200, 500, and 1000 μm. When the trabecular space height *z* is above 200 μm, most regions within the trabecular space have FSS values higher than 3 Pa, and when *z* reaches 1000 μm, the peak FSS in the trabecular space can reach about 15 Pa (Figure 4A), with the peak FSS showing a positive correlation with trabecular bone space height (Figure 4B). Secondly, four microcrack depths were set at 100, 50, 20, and 10 μm. It was found that the FSS at the microcracks is significantly lower than the FSS in the space, resulting in an inverted “U”-shaped distribution of FSS along the *X*-axis in the trabecular microstructure model (Figure 4C), but the peak FSS within the trabecular space was not affected by the depth of the trabecular microcracks. Lastly, four microcrack widths were set at 100, 50, 20, and 10 μm. The numerical simulation results for the four trabecular microcrack widths are similar to those for the depths, with FSS distributions along the *X*-axis showing low ends and high middle; that is, the shear stress at the microcracks is lower than that in the trabecular space (Figure 4E), and the peak FSS values within the trabecular space are almost the same (Figure 4F). This indicates that the depth and width of the trabecular microcracks do not affect the peak FSS within the trabecular space.

### 3.4. The FSS Within Microcracks of Trabecular Bone Exhibits a Gradient Distribution

**Figure 5:**
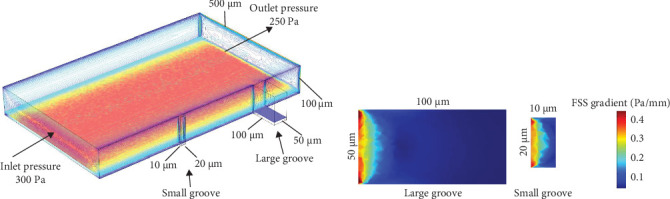
Gradient distribution of fluid shear stress in the microcracks of bone trabeculae. (A) Establishment of the finite element numerical model of the trabecular microstructure. (B) Distribution of fluid shear stress inside the microcracks of the bone trabeculae.

To investigate the distribution of FSS within microcracks in trabecular bone, this study established models of microcracks at various sizes. The large groove featured a depth and width of 100 μm and 50 μm, respectively, while the small groove had dimensions of 10 μm in depth and 20 μm in width (Figure 5A). Numerical simulation results indicated that the gradient range of wall shear stress inside the cracks was between 0 and 0.4 Pa/mm, with most areas within the large grooves showing a FSS gradient of less than 0.1 Pa/mm (Figure 5B). From the results of this study, we can analyze the presence of varying environments of FSS gradients within the microcracks of trabecular bone. Further research will focus on developing a gradient FSS device for studying the migration of living cells.

## 4. Discussion

In this paper, the pressure difference of four kinds of bone trabecular space was simulated by finite element method, which were 250 Pa, 200 Pa, 100 Pa, and 50 Pa, respectively (Figure 1), which includes the range of human blood pressure [40]. We found that the peak value of FSS in the same bone trabecular gap width was positively correlated with the pressure difference at both ends of the finite element numerical model (Figure 1A,B). There is evidence that osteoblasts and osteoblasts are not susceptible to biological responses under solid stress or FSS below physiological levels. The distribution of these stresses is influenced by both bone microarchitecture and the viscosity of the interstitial fluid [41–43]. Recently, we established a 3D finite element model using the real structure of the cancellous bone of the distal femur in mice. The fluid–structure coupling numerical simulation results show that the stiffness along the physiological load direction is significantly higher than that perpendicular to the physiological load direction [7]. More importantly, this study revealed that there was a “lower limit” line in the relationship between von Mises equivalent stress in the solid domain and FSS in the liquid domain at the solid–liquid interface of bone trabeculae; that is, the FSS there would not be lower than the corresponding threshold value for high solid stress values. However, for low solid stress values, the corresponding FSS will be distributed in a large range. These results indicate that there is no simple one-to-one correspondence between matrix stress and FSS for osteoblasts or osteoclasts attached to the surface of bone matrix, and it is necessary to focus on the regulation of local FSS on cell biological response, especially the effect of fluid flow on osteoclasts, which has received little attention before.

In order to study the distribution of local FSS, finite element models of six kinds of bone trabecular gap widths and four kinds of bone trabecular gap heights were established (Figure 3). On this basis, the effects of the depth and width of four kinds of microcracks on the peak value of FSS and the local FSS gradient are studied (Figures 4 and 5). When the trabecular gap width was reduced to 100 μm, the peak FSS in the gap was reduced by 28.57% compared with 800 μm (Figure 3C). The height of bone trabecular space is positively correlated with the peak value of FSS (Figure 4A,B). Other studies have found that there is a colocalization relationship between the bone remodeling area and the intrabone microinjury area, in particular, bone resorption usually occurs near the large scale microinjury but not near the small scale microinjury with a diffuse distribution [8–10]. Therefore, it is reasonable to speculate that osteoclast precursor cells sense the FSS gradient near the microinjury and migrate to the low FSS area inside the microinjury, gradually differentiate into mature osteoclasts, and finally initiate bone resorption. Figure 5A establishes the FSS model of the bone trabecular microdamage area. The FSS gradients in the areas with large cracks and small cracks can be seen from Figure 5B. It is found that the FSS gradients at the cracks range from 0 to 0.4 Pa/mm, and there are more areas at the large cracks where the FSS gradients are lower than 0.1 Pa/mm. There is evidence that osteoblasts and osteoblasts are not susceptible to biological response under solid stress or FSS below physiological level [41–43]. The important significance of this discovery is that it provides cell biological evidence of bone resorption under external force. Combined with the existing cell biological evidence of bone formation, it is expected to achieve accurate simulation of bone structure evolution based on the mechanical microenvironment around cells and finally crack and clarify the “Wolff law.”

One major advantage of this study is the use of finite element numerical models with real trabecular bone structural properties to calculate the distribution of local FSS, where the gradient of FSS inside the microcrack was analyzed, enabling a reliable theoretical basis for the subsequent construction of gradient FSS devices in vitro [7, 21, 22]. This approach significantly reduces computational workload, improves convergence, and allows for direct measurement of the FSS inside the trabecula bone, avoiding the need for accurate boundary conditions and mitigating the effects of fluid boundaries in local models [44, 45]. Consequently, it enables accurate quantification and investigation of flow velocity and wall FSS within trabecular bone. Furthermore, by establishing an idealized trabecular bone model, this study provides novel insights into the mechanical coupling, offering a new approach to evaluate FSS and the mechanical behavior of cells.

Firstly, this study has several limitations. Despite over a century of research, it is well-established that bone structures undergo dynamic structural adjustments in response to changes in external mechanical loads [46, 47]. Similarly, trabecular bone is a continuous structure, and local models that disregard the connectivity between bone regions lead to significant boundary effects due to the limitations in representing marrow boundaries. Consequently, while previous studies have provided valuable insights into the mechanical properties of the marrow–trabeculae system, the constraints imposed by local models significantly increase the error in the research outcomes.

Secondly, due to computational limitations, the fluid characteristics in this study are assumed to be homogeneous, whereas, in reality, marrow can more accurately be described as a colloidal suspension of cells [31]. To assess the effects of varying interstitial fluid velocity, bone trabecular structure, and microcrack size on the distribution of FSS within the trabecular bone space, the trabecular bone model is not based on its actual structure but instead uses real bone microstructure parameters to approximate the interstitial FSS distribution. Given the variability of bone structures, future research should aim to employ real bone structures for more accurate representation.

Finally, the environment between trabeculae is characterized by complex, tortuous geometry, and the marrow is assumed to exhibit laminar flow in this study. However, the model neglects the instantaneous changes in fluid behavior and also instead assumes a steady-state flow as literature [48]. Future research will focus on refining the model by incorporating more accurate boundary conditions and investigating fluid–solid coupling computations in intact bones.

Understanding the distribution of FSS within bones is essential for advancing our knowledge of bone metabolism and pathology. Continued advancements in simulation technology and experimental methods will enhance our ability to predict and modify bone responses to mechanical stimuli. In the next step, a new device will be developed based on the study.

## 5. Conclusion

This paper studies the FSS in bone microstructure regions by using a finite element numerical simulation model with trabecular bone as the research subject. The study considers various factors such as different inlet and outlet pressures, the width and height of trabecular gaps, and the width and height of microcracks in trabeculae. It also calculates the FSS gradient in the microdamage regions. The simulation results confirm that different inlet and outlet pressures as well as the width and height of trabecular gaps affect the peak FSS within the gaps and are positively correlated with the peak values of the trabeculae. Moreover, the FSS within the gaps increases exponentially with the increase in gap height. The variations in the width and height of microcracks do not affect the FSS within the gaps, and there is a gradient change of FSS within the bone microdamage. The findings of this study can provide a strong theoretical basis for the development of a living cell gradient FSS device.

## Figures and Tables

**Table 1:** Model parameters of bone trabeculae.

Model parameter	Values for bone trabecular structure
Length of model (μm)	1500
Width of the bone trabecular interstices (μm) [35]	1000, 800, 600, 400, 200, and 100
Height of the trabecular interstices (μm) [35]	1000, 800, 600, 400, 200, and 100
Microcracks of trabecular bone (μm) [36, 37]	Large groove featured (depth of 100 and width of 50)
	Small groove featured (depth of 20 μm and width of 10)
Pressure difference at the entrance and exit (Pa) [21, 24, 32]	250, 200, 100, and 50

## Data Availability

All data included in this study are available upon request by contact with the corresponding author.
